# Complex hydrocephalus (combination of communicating and obstructive type): an important cause of failed endoscopic third ventriculostomy

**DOI:** 10.1186/1756-0500-2-137

**Published:** 2009-07-16

**Authors:** Yad Ram Yadav, Gaurav Mukerji, Vijay Parihar, Mallika Sinha, Sanjay Pandey

**Affiliations:** 1 Neurosurgery unit, NSCB Medical College, Jabalpur, India; 2 Urology, Charing Cross Hospital, London, UK; 3 Radio diagnosis, NSCB Medical College, Jabalpur, India

## Abstract

**Background:**

Hydrocephalus can be classified as purely obstructive, purely communicating or due to combinations of pathologies (obstruction in addition to defective absorption). Endoscopic third ventriculostomy (ETV) as an alternative to shunt procedures is an established treatment for obstructive hydrocephalus. However, patients who have combination of pathologies (complex hydrocephalus) could result in failure of ETV in spite of a patent stoma. The aim of this study was to prospectively evaluate the incidence of complex hydrocephalus in patients with obstructive hydrocephalus who failed an endoscopic third ventriculostomy.

**Findings:**

Seventy one patients of obstructive hydrocephalus who underwent ETV in our institution were included in this study. Aetiology of hydrocephalus included congenital aqueductal stenosis in 42 and tubercular meningitis (TBM) in 29 patients. Failure of ETV was seen in 15 (21%) patients. These 15 patients included 6 (14.3%) from the congenital group and 9 (31.0%) patients from the TBM group. Iohexol CT ventriculography confirmed a patent stoma (suggesting a complex hydrocephalus) in 10 (66.7%) out of the 15 failed ETV cases. The incidence of complex hydrocephalus was more common in TBM group (8/29 patients, 27.60%) compared to congenital group (2/42 patients, 4.8%). The complex hydrocephalus patients with a patent ETV stoma were successfully managed by a lumbar peritoneal (LP) shunt.

**Conclusion:**

Ten out of the 71 patients (14%) with obstructive hydrocephalus who underwent an ETV had a complex hydrocephalus, which was the major (66.7%) cause for failure of ETV. Improving methods to detect the exact type of hydrocephalus pre-operatively could increase success rate of ETV and avoid an unnecessary operative procedure (ETV).

## Background

Hydrocephalus can be communicating, obstructive, or a combination of both. Understanding the basic pathology or the combination of pathologies leading to hydrocephalus in a given patient is essential to decide the best treatment option [1]. Patients with communicating hydrocephalus can be managed with a lumbar peritoneal (LP) shunt [2]. While, in purely obstructive hydrocephalus, an endoscopic third ventriculostomy (ETV) is a preferred option, as an alternative to ventriculoperitoneal (VP)/ventriculoatrial (VA) shunting [1,3]. In some cases, despite a functioning/patent stoma after an ETV, there is no clinical improvement and patients present with persistently raised intracranial pressure (ICP). In such cases, a combination of obstruction with defective absorption may be responsible for their clinical features. This entity with combination of pathologies is referred to as complex hydrocephalus. Complex hydrocephalus is an important cause of failed ETV despite of patent stoma. Complex hydrocephalus can be further sub-classified as temporary or permanent based on whether the defective absorption and/or defective permeation of CSF through subarachnoid space (SAS) is transient or persistent [1,4,5]. Repeated Lumbar Punctures could solve the problem if it is due to the temporary pathology [4]. A LP shunt or VP shunt will be required in patients with failed ETV with patent stoma having persistent defect in CSF hydrodynamics [1].

The entity of complex hydrocephalus has been previously described [1,4,5], but none of the studies has reported its incidence. In this study, we evaluated the incidence of complex hydrocephalus in patients with obstructive hydrocephalus who failed an endoscopic third ventriculostomy.

## Materials and methods

In this prospective study, patients with obstructive hydrocephalus who underwent ETV in our institution during a two year period from July 2005 to June 2007 were included. The study was approved by the ethics committee of NSCB Medical College. Written consent was obtained from all the patients or their legal representative. A detailed history was taken and a thorough physical examination was performed in all the cases. The diagnosis was confirmed by a CT scan pre-operatively in all the patients. Due to resource constraints, MRI scan could be done in 45 patients only.

ETV was performed using the standard procedure [6]. Stoma of 5 mm or more was made in all cases. The patients were evaluated postoperatively by CT and/or MRI scans. Complications such as infection, CSF leak and failure of procedure were assessed. ETV was considered clinically successful when anterior fontanelle was depressed or flushed to the adjoining scalp (in infants) and/or patients improved clinically.

**Figure 1:**
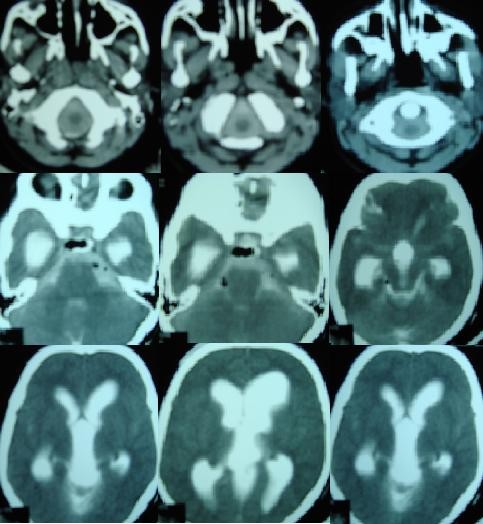
**Patent stoma in patient with failed ETV**. Iohexol CT ventriculography of failed endoscopic third ventriculostomy patient showing dilated lateral and third ventricles with good amount of dye in basal cisterns and subarachnoid space at atlas region.

Repeated lumbar punctures were done for 15 post operative days to relieve pressure in patients with persistent raised ICP. ETV was considered a failure when there was persistent or progressive dilatation of ventricles with persistently raised ICP. Iohexol ventriculography was performed in all failed ETV cases. A patent stoma was confirmed when good amount of dye in basal cisterns were observed (Figure 1). LP shunt was performed in cases with patent stoma and persistently raised ICP for more than 10–15 days even after 3–5 lumbar punctures. Repeat ETV was done in patients with blocked stoma. Repeated lumbar punctures were done in patients with CSF leak. LP shunt was done when the leak did not stop after repeated lumbar puncture over 7–10 days time. Repeat skin sutures were also applied if needed.

## Results

### Patient population

A total of 71 patients with obstructive hydrocephalus underwent ETV during the period from July 2005 to June 2007. Age of the patients ranged from 3 months to 76 years. Fifty-two patients (73.2%) were of the age group 18 years and below. There was no difference in the incidence of complex hydrocephalus in various age groups. Aetiology of hydrocephalus included congenital aqueductal stenosis in 42 and tubercular meningitis (TBM) in 29 patients.

### Complications and Outcome of ETV

Follow up ranged from 3 to 27 months with an average of 14 months. The procedure was considered successful with significant improvement of symptoms in 56 (78.8%) patients. Nine patients developed CSF leak. The leak stopped spontaneously in 5 cases in 5–7 days. One of our patients developed transient diabetes insipidus, which resolved of its own.

### Failure of ETV and Incidence of Complex hydrocephalus

Failure of ETV was seen in 15 (21%) patients. These included 6 (14.3%) from the congenital group and 9 (31.0%) patients from the TBM group. None of the patients with failed ETV developed postoperative infection. Iohexol CT ventriculography confirmed a blocked stoma in 6 of the 15 ETV failure cases. Repeat ETV was done in all 6 cases with blocked stoma, out of these 5 were successful. The remaining patient who failed following repeat ETV had a patent stoma confirmed by iohexol ventriculography.

Thus, a patent/functioning stoma was demonstrated in 10 (66.7%) out of the 15 failed ETV cases suggesting a combination of pathologies i.e. defective absorption along with obstruction (complex hydrocephalus), giving an incidence of 14% in our series of 71 obstructive hydrocephalus patients. The incidence of complex hydrocephalus was more common in TBM group (8/29 patients, 27.60%) compared to congenital group (2/42 patients, 4.8%). The complex hydrocephalus was responsible for 2 out of 6 (33.3%) and 8 out of 9 (88.9%) failed ETV patients of congenital and TBM hydrocephalus groups respectively.

All the complex hydrocephalus patients were successfully managed by a lumbar peritoneal (LP) shunt.

## Discussion

The conventional treatment of obstructive hydrocephalus is CSF diversion [7,8], usually by performing a shunt procedure. Shunts divert the CSF flow into extracranial body cavities such as the peritoneal cavity (VP shunt). Endoscopic third ventriculostomy (ETV which creates a flow of CSF endoscopically from the third ventricles to the subarachnoid space, is an alternative form of CSF diversion [3]. It is a minimally invasive procedure and has established itself in the management of obstructive hydrocephalus. The reported success rate of ETV is usually in the range of 60 to 80% [8,9]. The success rate of ETV in our series was 78.8%. Previous studies have evaluated various risk factors for the failure of ETV, such as cause of hydrocephalus, presence and function of shunts, history of shunt revisions or infections, symptoms, preoperative imaging, infection, etc [8,10]. In this study, we have identified complex hydrocephalus as a major (66.7%) cause for failure of ETV. The persistently high ICP observed in this group of patients despite a patent stoma and relief of obstruction is probably related to the slow permeation of CSF through the SAS, defect in absorption of CSF or both.

Complex pathologies in hydrocephalus have been reported in other series [1,4,5,7]. Beni-Adani et al. [1] categorized infants with active hydrocephalus into the four groups along the spectrum of communicating verses obstructive hydrocephalus. Of the four groups, group 2 and 3 were patients with an obstructive component together with a persistent or temporary absorptive component respectively [1]. Nishiyama et al. [7] also suggested that obstructive hydrocephalus in the very young population may be rather a combination of obstructive and absorptive problem. However, none of the previous studies have reported the incidence of complex hydrocephalus. In our study, 10 of 71 patients (14%) had complex hydrocephalus.

The failure rate of ETV was higher in TBM group (31.0%) compared to the congenital group (14.3%). Similar results have been observed by other authors [3,11-15]. It has been proposed that the higher risk of failure in TBM group could be attributed to high chances of complex pathologies due to obliteration of cerebrospinal fluid pathways [12,15,16]. In support of the hypothesis, we observed a higher incidence of complex hydrocephalus in TBM group (27.60%) and was responsible for majority (88.9%) of failed ETV cases in the group.

Postoperative high ICP has been observed following ETV by various authors [4,5,7]. It was seen that CSF dynamics convert from a shunt-dependent state to a shunt-independent state within few weeks following ETV in majority of patients with non-communicating hydrocephalus [7]. The intraventricular pressure does not decrease rapidly in some cases. Cerebrospinal fluid absorptive capacity or CSF circulation through the SAS may show further improvement several months after ETV [7]. Persistent symptoms after ETV were also observed by Cinalli et al. [4]. They suggested that a cycle of one to three lumbar punctures should be performed in patients who remain symptomatic and who show increasing ventricular dilatation after ETV prior to assuming a failure of ETV and an extra cranial cerebrospinal fluid shunt is implanted [4]. In our study, all complex hydrocephalus patients had persistently raised ICP for more than 10–15 days even after 3–5 lumbar punctures. They were successfully managed by a LP shunt.

## Conclusion

The incidence of complex hydrocephalus in patients diagnosed with obstructive hydrocephalus was 14%, which was the major (66.7%) cause for failure of ETV. Proper pre-operative understanding of CSF hydrodynamics to detect the type of hydrocephalus is essential to decide the best treatment option, improve postoperative outcome and avoid an unnecessary operative procedure.

## Competing interests

The authors declare that they have no competing interests.

## Authors' contributions

YRY has made substantial contribution to conception and design of the study, performed the surgical procedures, was involved in acquisition, analysis and interpretation of data, writing and revising of the manuscript. GM made substantial contribution in writing and critically revising the manuscript. VP and MS were involved in clinical assessment of patients, acquisition, analysis and interpretation of data and drafting of the manuscript. SP contributed to design and interpretation the radiological investigations of the patients. All authors have read and approved the manuscript.
